# The new patent regime: Implications for patients in India

**DOI:** 10.4103/0019-5545.31520

**Published:** 2007

**Authors:** Chittaranjan Andrade, Nilesh Shah, Sarvesh Chandra

**Affiliations:** National Institute of Mental Health and Neurosciences, Bangalore - 560 029, India; * LTM Medical College and Hospital, Sion, Mumbai - 400 022, India; ** National Institute of Mental Health and Neurosciences Bareilly, UP, India

Pharmaceutical companies spend billions of dollars on research. It is estimated that, of every thousand potential drugs screened, only 4-5 reach clinical trials and only one is actually approved for marketing. Pharmaceutical companies patent the drugs that they develop and thereby obtain exclusive marketing rights; the costs of research and the profits due to the shareholders are recovered through appropriate pricing mechanisms from the patients who receive the patented drugs.

Internationally, drug patents and the exclusive marketing rights associated therewith are awarded for a period of 20 years; during this time, no other drug company is allowed to manufacture or market the same drug. After the patent expires, other companies are permitted to manufacture and market the drug; their brands are known as generic versions.

In the early 1970s, the Indian Patents Act was passed under the Indira Gandhi government to permit greater access of medicines at lower rates to the poor in the country. According to the Act, process patents but not product patents would be recognized. Expressed otherwise, India would award patents not to individual drugs but to the process whereby the drug was manufactured. This allowed Indian drug companies to manufacture the same drug using other processes (this is otherwise known as reverse engineering). As the Indian companies incurred little expenditure on research and development of new drugs, it became possible to make new drugs available to the country at affordable rates.

As India sought to improve its presence in the global market, it became clear that it could no longer protect domestic consumers in its patent policy. India is a member of the World Trade Organization. India therefore requires a new patent law to fulfil its obligations under the trade-related aspects of intellectual property rights (TRIPS). India became a member of the Paris convention and signed the Patent cooperation treaty with effect from December 7, 1998. Since then, amendments to the Patent Act were enacted in April 1999 and May 2002. The third amendment became due. The necessary bill to make the Indian Patents Act TRIPS-compliant was supposed to have been tabled during the 2004 winter session of Parliament; instead, an ordinance was passed on December 26, 2004, which came into effect on January 1, 2005. This ordinance modified the Indian Patents Act. This ordinance was itself modified and the Patents (Amendment) Bill was passed by the Lok Sabha and Rajya Sabha on March 22 and March 23, 2005, respectively. The President signed the bill on April 5, 2005, making it an Act of Parliament.

## AT PRESENT, THE SCENARIO IN INDIA IS AS FOLLOWS

1. India will respect product patents. However, the patents so respected will only be those issued in India.
2. Product patents will be respected for a period of 20 years from the time of application and not from the time of grant of the patent. About ten thousand applications for patents were pending with the government in 2005; these date back to 1995 and are designated as mailbox applications. It will take several years to screen all the applications and award patents as appropriate. This will increase the breathing space for Indian pharmaceutical companies and Indian consumers.
3. New applications for patents will also be processed; again, the grant of patent will be for 20 years from the date of application. This is in accordance with the Patent Cooperation Treaty which India has signed, which will make it possible for a new invention to be simultaneously patented in a large number of countries.
4. Other agencies interested in the product will be provided an opportunity to oppose the grant of patent. Both pre-grant and post-grant opposition will be entertained. In the December 2004 ordinance, pre-grant opposition had been emasculated to a written application with no further representation allowed on the part of the opposer; in contrast, under the previous patent act, pre-grant opposition was a more powerful procedure with the opposer having a right of audience to the proceedings involved in the grant of patent. With the new Patents Act of 2005, pre-grant opposition has been strengthened: more time has been allowed and the opposer has been given the right to be a party to the proceedings.
5. Even though the patent will be awarded with retrospective effect from the date of application, the implementation of the patent will only be with prospective effect. Thereby, generic versions of a drug will need to be withdrawn only after a patent is awarded and the companies manufacturing and marketing the generic drugs will not be retrospectively liable for having manufactured and marketed the drug. Furthermore, companies manufacturing products patented between 1995 and 2005 will be allowed to continue to do so after paying a reasonable royalty to the patent holder.
6. Companies sometimes resort to evergreening to extend the duration of their hold of a patent. Evergreening refers to the making of minor modifications in a drug structure or formulation. The December 2004 ordinance passed by the Indian government did not address evergreening. However, in the Patents Act of 2005, the definition of patentability was modified to prevent evergreening. As an example, this could mean that once-weekly fluoxetine and escitalopram would likely not be granted fresh patents to extend the marketing rights of the patent holders of fluoxetine and citalopram, respectively.
7. Fresh patents will not be granted for new indications for drug use; this was not explicitly prohibited in the December 2004 ordinance, but has been clarified in the Patents Act of 2005.

## PROBLEMS THAT INDIAN PATIENTS MAY FACE

1. When the mailbox applications are cleared and patents awarded, newly-introduced generics in the Indian market may have to be withdrawn. This, for example, is why Indian brands of tadalafil have disappeared from the shelves. And, newer antipsychotic, antidepressant, antiepileptic and other drugs will be permitted to be marketed only by the patent holder. Costs to the patient will then inevitably rise. This scenario is feared but is by no means certain to occur as the international patents for almost all currently available drugs had been awarded before January 1, 1995, the cut-off date.
2. New drugs that emerge in the international arena will be available to Indian patients only from the patent holder. Again, the cost is almost certain to be high.

## A SMALL CONSOLATION

A small consolation is that the bulk of the neuropsychiatric pharmacopoeia is out of patent and will remain available in the generic form.

## DEFENCES AGAINST EXORBITANT PRICING AND UNAVAILABILITY

1. Tie-ups: Multinational drug companies have a weak presence in India: their drug basket is small, their marketing structure is weak and their domestic operations are limited. Multinational companies may need to tie-up with Indian companies for effective marketing. This may result in greater affordability to Indian patients. There is already evidence that Indian and multinational companies are exploring opportunities for mutual benefits. It is, however, unlikely that new drug prices will be as low as currently enjoyed by the Indian public.
2. Compulsory licensing: The Indian government has reserved the right for compulsory licensing; that is, providing Indian companies the privilege to manufacture and market a drug even before the expiry of the patent held for that drug. Compulsory licensing will be resorted to if the patent holder does not make the drug available to Indian patients or if the cost to Indian patients is too high. Compulsory licensing for export will also be resorted to, on similar grounds, to supply drugs to poor countries to meet their acute public health problems as per the TRIPS agreement of the Doha Declaration on Public Health.
  By way of example: the Brazilian Government recently announced that it would break the patent on several retroviral drugs to prevent the financial collapse of its successful public health program which provided free medication to HIV/AIDS patients.
  Article 31 of the TRIPS agreement provides for compulsory licensing without the authorization of the patent holder in the case of a national emergency or other circumstances of extreme importance or in cases of public, noncommercial use. This idea is also embodied in Section 92 of the Indian Patents Act of 1970. It is, however, uncertain that circumstances will arise which will make the Indian Government resort to compulsory licensing for psychotropic medication.
  If compulsory licensing is to succeed, some absurdities in the existent Patent Act require to be removed. One absurdity is that a compulsory license cannot be awarded during the first three years of the grant of a patent. Another absurdity is that the applicant for a compulsory license is required to state the nature of his interest in the matter and the existing patent holder is allowed to oppose the grant of the application. While this is correct on the grounds of natural justice, it defeats the needs of emergency licensing. A third absurdity is that compulsory licensing is possible only for drugs which are patented in the country and not for those which are patented elsewhere. Pharmaceutical companies can therefore avoid compulsory licensing if they do not apply for a patent in India.
  According to the provisions of the Patents Act of 2005, generic versions of patented drugs will be permitted to be manufactured and exported under a compulsory license to meet the major health needs of underdeveloped countries if the concerned countries issue a notification that the drug is required for the purpose.
3. Price control: The Indian Government has a list of drugs under price control. The exercise of this option may protect patients against exorbitant pricing. However, this option is unlikely to be exercised for newer psychotropic drugs unless the drugs have dramatic health benefits.

## INDIRECT BENEFITS OF THE NEW PATENT REGIME

1. It will force the Indian pharmaceutical sector into greater efforts in research and development. Many of the pharmaceutical majors in India have already made large outlays in this area and have even applied for patents, though not necessarily for psychotropic drugs or even chemicals with therapeutic potential.
2. Outsourcing of laboratory research and clinical trials to India will increase, thereby facilitating the domestic processes for the approval of the marketing of a new drug. Even more importantly, outsourcing to India will lower research costs, thereby reducing the costs which will have to be recovered through pricing mechanisms. Finally, even bulk drug manufacture may be outsourced to India, which would further reduce the costs of the marketed product.
3. Small companies, many of which manufacture and market generic drugs of doubtful quality, will fold up.
4. Competition will eventually change from brand vs brand to drug vs drug.

## UPDATE ON EVERGRENING

At present, there is a strong lobby trying to persuade the government to allow evergreening; that is, the patenting of molecules which differ slightly from the parent molecule. The argument is that molecules are patented very early during the process of drug discovery, but unique clinical characteristics or benefits are not discovered until much later, when clinical trials are conducted, if at all. Therefore, it is unreasonable to ask that unique characteristics of a slightly altered molecule be described at the time of the application for the patent, itself. Evergreening is not necessarily a disadvantage to India. For example, if evergreening is permitted, Indian companies may be able to develop and patent incremental advances on patented drugs.

A government-appointment committee on patent laws,[1] headed by R. A. Mashelkar, a former chief of the Council for Scientific and Industrial Research, favored the grant of patents to all incremental innovations made to a drug, but not to frivolous evergreening. The report was widely interpreted to permit most forms of evergreening. The report also favored the grant of patents on microorganisms to make the Indian Patents Act TRIPS-complicant. The report was withdrawn in mid-February, 2007, after it was discovered that a part of the report was lifted, without acknowledgement and verbatim, from a paper published by a UK-based organization which had been funded by the pharmaceutical industry. In March, 2007, the Government requested the Mashelkar committee to revise and resubmit its discredited report.

On a related note, the patents act does not define how unique the new molecule must be; therefore, an element of subjectivity enters the decision-making process for the grant of a patent. In this context, the pharmaceutical industry is concerned that the officials involved in the grant of patents may not be sufficiently qualified to understand the nuances in molecular behavior that justify novelty and hence the grant of a patent.

## UPDATE ON POSSIBLE PRICE CONTROL FOR PATENTED DRUGS

On January 26, 2007, the Union Ministry of Chemicals and Fertilisers announced that it was considering the formation of a committee which would suggest a system of price negotiation for patented drugs so that such drugs could be made available at an affordable price within the ambit of the National Pharma Policy. Without negotiated pricing, these drugs would not be given marketing rights in India. The committee would be headed by a Director (Chemicals) and would have representatives from all concerned ministries, including the ministries of health and commerce. The recommendations of the committee, if approved, would need to be made a legal requirement through an amendment of the Drugs and Cosmetics Act. A 7-member committee has now been set up.

## NOTE ON GENE AND MICROORGANISM PATENTS

The USA allows gene patents; therefore, individuals or private organizations can own the intellectual rights on genes that determine health and disease. This will allow such individuals or organizations to permit or deny others the permission to research or even test for these genes or diseases. This adversely impacts upon medical progress and even individual healthcare. At present, over 20 human pathogens are privately owned, including Hemophilus influenzae and the Hepatitis C virus. The scandalous implications were well-discussed by Crichton.[2]

## CONCLUDING NOTES

Several ministries and departments are involved in the areas discussed in this report. The most important is The Controller of Patents, Government of India. This is the authority which will screen applications for patents, award patents and award compulsory licenses.

## RECOMMENDED READING

1. Qaiser M, Chandran MP. Patent holder deserves monopolistic rights. Indian J Pharmacol 2006;38:73-5.
2. Thawani V, Gharpure K, Thawani M. Patent laws must be in the national interest. Indian J Pharmacol 2006;38:70-2.
